# The Slaughter Cure for Tuberculosis

**Published:** 1900-11

**Authors:** 


### SELECTIONS.

### THE SLAUGHTER CURE FOR TUBERCULOSIS.

A PERUSAL of several articles in some of the veterinary journals on tuberculosis and the tuberculin test would lead one to believe that the thirst for blood said to be latent in every human being has broken forth in the veterinary profession Nero-like. Some of these gentlemen would consign to the pyre all reactors to the tuberculin test. Pure bred or scrub, fat or lean, good milker or poor milker, they all must go. It is difficult to understand the reasoning which leads to such conclusions, unless it is scientific teaching run mad. A more sordid explanation presents itself, but even on this basis the dullest-minded veterinarian should see that he is not subserving his own personal interests by the advocacy of such extreme measures. Of course, the institution of such a sweeping policy of slaughter would provide temporary jobs for the vets, but it would kindle such resentment in the minds of plundered stockmen as would cost the veterinary profession dearly in the end. These extremists are sowing the wind and they will reap the whirlwind. They have already alienated hundreds of stockmen who will lose animals by disease rather than employ the men who have advocated the slaughter of their cattle. On the most selfish basis these unrestrained vets are throwing boomerangs. They should have a care. The worm is already turning. A shotgun quarantine is not an impossible thing. They have run amuck with tuberculin squirt-gun and pole-axe among the herds of farmers until patience has ceased to be a virtue, and the wave of rising retribution will engulf them with dire results. Farmers will no longer submit to such highway robbery under guise of law. This much on the sordid side.

But all this destruction of valuable property by over-zealous veterinarians has been done in the name of science, and yet the leading scientists, the men who investigate and reason, not the crowd that follows blindly with never a thought of its own, do not recommend the slaughter course. Bang and Nocard, who have devoted the most careful study to the question, are more moderate and sensible in their teachings, and their views, along with the experience of Russell at the Wisconsin station (which has induced him to change his position into which he was led by blind leaders of the blind), show that slaughter based on the tuberculin test is both irrational and wasteful. It is nothing but a doctrine of extremists, and extremists in medicine have oftener been wrong than right.

Stockmen have suffered their share at the hands of such unbalanced scientists. The demand for the indiscriminate slaughter of all lumpy-jawed cattle has not faded from memory, and hardly had this rank heresy been uprooted when the tuberculin craze intrudes. Tuberculin is in many cases a useful diagnostic agent in proper hands and at the proper time, but there its usefulness ends. It does not indicate the extent of disease in any particular case; that must be decided to a great extent by the clinical symptoms backed up subsequently by careful *post-mortems*. Ample evidence of the fallibility of the test has been brought to light on the Continent, in Britain and in this country, and no one longer ventures to dispute the fact that the worst cases of tuberculosis, those which may really prove dangerous under favoring conditions, are ofttimes wholly undetected by the tuberculin test.

When tuberculosis is scheduled as a contagious disease in the human family, along with smallpox, diphtheria, and the like, then perhaps we may listen with some patience to the slaughter proposition. But that time will never come. The war on the stockmen's interests has proceeded on the absolutely false basis that tuberculosis is a contagious disease. If it were such a disease as smallpox or scarlet fever the whole human race would have been wiped off the face of the earth centuries ago. Not a man among the "scientific" matadors who are brandishing the pole-axe has dared deny that proposition. The disease *may* be transmitted if the conditions are favorable for the tubercular bacilli to find suitable ground for growth. *But the healthy human system affords no habitat for the germs of tuberculosis.* This is the fundamental fact of this disease; it is the impregnable truth in the presence of which the falsehood of "contagion" rattles unspoken in the throat of the special pleader. But grant them their false premise; concede tuberculosis to be a contagious disease as the term is generally understood: what then? Kill all reacting cattle—and *then apply the pole-axe to all consumptive people!* In this way only can the disease be eradicated. And even such impossible methods would prove absolutely futile, as the air is full of the germs of tuberculosis. Dirt scraped from a car-window has revealed them by the thousand under the microscope. Churches and theatres and other places of public gatherings hold the bacilli in carpets and cracks and corners. The dust of our streets may be laden with them. They may be encountered everywhere. But they are harmless to the healthy system and only find lodgement where conditions of the mucous surfaces are peculiarly adapted to their growth by predisposition or disease. Not even by slaughter of cattle and consumptives could the disease be eradicated if it were a contagious disease as such diseases are understood.

The absolute folly of the slaughter crusade, the essential wickedness of it, thus stands boldly forth. The only wonder is that stockmen have suffered it thus far.—From the *Breeders' Gazette*, Chicago, October 24, 1900.

[See editorial comment on the above article in this issue.]

